# Oxidative Stress Causes Enhanced Secretion of YB-1 Protein that Restrains Proliferation of Receiving Cells

**DOI:** 10.3390/genes9100513

**Published:** 2018-10-22

**Authors:** Andrea Maria Guarino, Annaelena Troiano, Elio Pizzo, Andrea Bosso, Maria Vivo, Gabriella Pinto, Angela Amoresano, Alessandra Pollice, Girolama La Mantia, Viola Calabrò

**Affiliations:** 1 Dipartimento di Biologia, Università degli Studi di Napoli, Federico II, 80126 Napoli, Italy; andreamaria.guarino@unina.it (A.M.G.); annaelena.troiano@unina.it (A.T.); elipizzo@unina.it (E.P.); andrea.bosso@unina.it (A.B.); maria.vivo@unina.it (M.V.); apollice@unina.it (A.P.); lamantia@unina.it (G.L.M.); 2 Dipartimento di Scienze Chimiche, Università degli Studi di Napoli, Federico II, 80126 Napoli, Italy; gabriella.pinto@unina.it (G.P.); angamor@unina.it (A.A.)

**Keywords:** cold shock proteins, stress granules, oxidative stress, protein secretion

## Abstract

The prototype cold-shock Y-box binding protein 1 (YB-1) is a multifunctional protein that regulates a variety of fundamental biological processes including cell proliferation and migration, DNA damage, matrix protein synthesis and chemotaxis. The plethora of functions assigned to YB-1 is strictly dependent on its subcellular localization. In resting cells, YB-1 localizes to cytoplasm where it is a component of messenger ribonucleoprotein particles. Under stress conditions, YB-1 contributes to the formation of stress granules (SGs), cytoplasmic foci where untranslated messenger RNAs (mRNAs) are sorted or processed for reinitiation, degradation, or packaging into ribonucleoprotein particles (mRNPs). Following DNA damage, YB-1 translocates to the nucleus and participates in DNA repair thereby enhancing cell survival. Recent data show that YB-1 can also be secreted and YB-1-derived polypeptides are found in plasma of patients with sepsis and malignancies. Here we show that in response to oxidative insults, YB-1 assembly in SGs is associated with an enhancement of YB-1 protein secretion. An enriched fraction of extracellular YB-1 (exYB-1) significantly inhibited proliferation of receiving cells and such inhibition was associated to a G2/M cell cycle arrest, induction of p21WAF and reduction of ΔNp63α protein level. All together, these data show that acute oxidative stress causes sustained release of YB-1 as a paracrine/autocrine signal that stimulate cell cycle arrest.

## 1. Introduction

Oxidative stress is linked to a number of chronic diseases including diabetes, neurodegenerative and cardiovascular diseases, cancer, and aging [1]. The cold-shock Y-box binding protein 1 (YB-1) is involved in stress response [2] and chronic inflammation [3,4,5]. YB-1 is a member of the evolutionarily conserved cold shock domain (CSD) proteins and was first identified as a DNA binding protein interacting with the Y-box motif present in the major histocompatibility complex class II gene [6]. YB-1 binds to DNA and RNA [7] and it is involved in the transcriptional and translational control of many biological processes including cell proliferation and migration [8,9,10,11]. Moreover, YB-1 protein is upregulated in many types of human cancers including, breast, prostate, ovarian and melanoma [12,13].

Human YB-1 contains 324 amino acids [14]. Its structure can be subdivided into three domains namely N-terminal, cold shock (CS) and C-terminal domain. The short N-terminal domain, named A/P, contains 1–51 residues and is rich in alanine and proline. The central part of YB-1 is a CS domain conserved from bacteria to human (52–129 residues) [9]. The long C-terminal domain (130–324 residues) contains positive and negative charged clusters of amino acids.

Accumulating evidence links YB-1 to the cell response to oxidative stress [2] and DNA damage [15,16,17]. More specifically, following acute oxidative stress, YB-1 localizes to cytoplasmic stress granules (SGs) where it participates in a pro-survival mRNA translational reprogramming [2]. Cytosolic YB-1 can also be found in P-bodies [18], TIA-containing SGs, but its precise role in these cytoplasmic structures remains to be defined [2,19,20,21].

Following DNA damage, YB-1 translocates to the nucleus to oversee DNA damage repair mechanisms [16,17]. Indeed, genotoxic stress triggers a limited cleavage of YB-1 by the 20S proteasome with the consequent accumulation of a 36 kDa truncated form of YB-1 (amino acids 1–219) in the nucleus [22]. Truncated YB-1 associates with Mre11 and Rad50 in DNA repair complexes [16]. Nuclear shuttling of YB-1 also takes place during cancer growth and upon activation of pro-survival signaling cascades such as PI3K/AKT, RSK, Ras/MAPK and PKC and is triggered by phosphorylation at serine 102 of YB-1 [23,24,25,26,27].

Recent data show that YB-1 can also be secreted after cytokine challenge by mesangial and immune cells [28] and is increased in sera from sepsis and tumor patients [29,30,31]. Here, we focused our studies on secreted YB-1 and found that oxidative stress enhances YB-1 secretion. Finally, using extracellular YB-1 (exYB-1) we provide some evidence of a role of exYB-1 as an autocrine/paracrine signal inducing cell cycle arrest.

## 2. Materials and Methods

### 2.1. Plasmids and Chemicals

The expression construct 5xMyc-YB-1 was previously described [32]. Plasmid pcDNA 3.1B, used as a control, was purchased by Thermo-Fisher Scientific (Waltham, MA, USA).

Sodium (meta)arsenite (NaAsO_2_) (S7400 Sigma-Aldrich, Saint Louis, MO, USA); hydrogen peroxide (H1009 Sigma-Aldrich, Saint Louis, MO, USA) were used to perform oxidative stress. Trichloroacetic acid (TCA) (T4885 Sigma-Aldrich, Saint Louis, MO, USA) was used to extract proteins from cell culture medium.

### 2.2. Cell Culture

Human embryonic kidney cells (HEK293T), spontaneously immortalized keratinocytes from adult skin (HaCaT), and human colorectal adenocarcinoma cells (CaCo-2), were purchased from Cell Line Service (CLS, Eppelheim, Germany) and grown as previously described [33].

To increase HEK293T adhesion to glass/plastic surfaces, plates and slips for immunofluorescence were treated with poly-D-lysine 0.1 mg/mL (P7405, Sigma-Aldrich, Saint Louis, MO, USA) before seeding cells.

### 2.3. Immunofluorescence Microscopy

Confocal immunofluorescence was performed as previously described [34]. All images were acquired using a Carl Zeiss LSM700 (63× oil immersion objective). Image processing and analysis were performed with Fiji (ImageJ version 2.0) software.

### 2.4. Cell Viability Assays

Cell viability was determined by the crystal violet assay (CVA) method, through a (3-(4,5-dimethylthiazol-2-yl)-2,5-diphenyl-tetrazolium bromide) MTT based assay, or through trypan blue count as previously described [35].

Cells were seeded in 96-well plate for MTT (8 × 10^3^ cells), in 35 mm dishes (2.5 × 10^5^ cells) for CVA and in 12-well plates (1.5 × 10^5^ cells) for trypan blue.

The day after plating, cells were treated or not. For crystal violet, dye uptake was measured at 570 nm using a Beckman Coulter spectrophotometer (DU730). Cell viability was calculated as dye intensity and compared with untreated samples.

For MTT, the optical absorbance was determined at 570 nm using an iMark microplate reader (Bio-Rad, Hercules, CA, USA). For trypan blue count, after treatment cells were gently rinsed with 1× Phosphate-buffered saline (PBS), trypsinized and collected. An aliquot was diluted 1:1 with trypan blue (Sigma-Aldrich, Saint Louis, MO, USA).

### 2.5. Cell Cycle Analysis

A total of 3 × 10^5^ cells were seeded and let grow for 24 h; then, cells were serum starved for 24 h to achieve synchronization; the day after, cells were treated with recombinant YB-1 (rYB-1) or Conditioned Culture Medium derived YB-1 (CCM-YB-1) or left untreated for 24 h. Cells were then trypsinized, collected in PBS with the addition of 1% FBS, 0.25 mM ethylenediaminetetraaacetic acid (EDTA) in PBS and centrifuged at 1200 rpm, 4 min, 4 °C; for each sample the same number of cells (1 × 10^5^) was processed. The cell pellet was resuspended in methanol, incubated on ice for 20 min and centrifuged at 1200 rpm, 5 min, 4 °C. After a wash in PBS, the pellet was incubated in PBS, containing RNase A (Thermo-Fisher Scientific) 100 μg/mL for 20 min at RT. Propidium iodide (Sigma-Aldrich, Saint Louis, MO, USA) was then added at a concentration of 50 μg/mL for 30 min at 4 °C. Cell cycle analysis was performed on the BD Accuri C6 flow cytometer (BD Biosciences, San Jose, CA, USA).

### 2.6. Cell Proliferation Analysis

A total of 6 × 10^4^ HaCaT and CaCo-2 cells were seeded in 12-well plate, while HEK293T were seeded on pre-treated 12-well; cells were serum starved for 24 h; after starvation, recombinant or CCM-YB-1 were added at different concentrations. Every 24 h cells were gently rinsed with 1× PBS, trypsinized and counted. The count was confirmed by Scepter 2.0 analysis (Millipore, Burlington, MI, USA) as previously described [36].

### 2.7. Transfections

Cells were transfected using Lipofectamine 2000 (Life Technologies, Carlsbad, CA, USA) according to the manufacturer’s recommendations. Briefly, cells were seeded at 70–80% confluence (1.5 × 10^6^) in 100-mm dishes and transiently transfected with different concentrations of plasmid (from 800 ng up to 1.5 μg).

YB-1 transient silencing was carried out with IBONI YB-1 small interfering (siRNA) pool (RIBOXX GmbH, Radebeul, Germany) and RNAiMAX reagent (Life Technologies, Carlsbad, CA, USA), according to the manufacturer’s recommendations. Cells were seeded at 70–80% confluence (1.5 × 10^6^) in 100-mm dishes and transiently silenced with IBONI YB1-siRNA at 100 nM final concentration.

All Star Negative Control siRNA, provided by Quiagen (Hilden, Germany), was used as negative control. YB-1 guide sequences:

5′-UUUAUCUUCUUCAUUGCCGCCCCC-3′; 5′-UUAUUCUUCUUAUGGCAGCCCCC-3′; 5′-UUCAACAACAUCAAACUCCCCC-3′; 5′-UCAUAUUUCUUCUUGUUGGCCCCC-3′.

### 2.8. Antibodies

Primary antibodies: anti-YB-1 raised against the region 1 to 100 of YB-1 protein (12148 Abcam, Cambridge, UK); anti-PABP1 (poly(A)-binding-proteiin 1) (clone 10E10, Sigma-Aldrich); anti-Myc (SAB21084786, Sigma-Aldrich, Saint Louis, MI, USA); anti-cyclin D1 (GT8912, Genetex, Irvine, CA, USA); anti-cyclin A2 (GT2547, Genetex, Irvine, CA, USA); anti-GAPDH (6C5 Santa Cruz Biotechnology, Dallas, TX, USA); anti-PARP1 (Cell Signalling, Danvers, MA, USA); anti-RNH1 (436–450, Sigma-Aldrich, Saint Louis, MO, USA) anti-β-tubulin (H-235, Santa Cruz Biotechnology, Dallas, TX, USA); anti-actinin (AT6/172, Abcam, Cambridge, UK); anti-p21 WAF (1D21 Cell Signaling, Danvers, MA, USA); anti-∆Np63α (4A4) (sc-8431 Santa Cruz Biotechnology, Dallas, TX, USA).

Secondary fluorescent antibodies: Alexa Fluor 488 anti-rabbit (Thermo-Fisher Scientific, Waltham, MA, USA); Cy3 anti-mouse (Jackson ImmunoResearch, Cambridge, UK); DAPI (Sigma-Aldrich, Saint Louis, MO, USA).

### 2.9. Immunoblot Analysis

Immunoblots were performed as previously described [37,38].

For total protein extraction and nuclear-cytoplasmic fractionation, cells were seeded at 60% confluence (1.5 × 10^6^ cells) in 100-mm dishes. Then, 24 h after seeding, cells were treated or left untreated; cell lysates were fractionated to obtain total, cytoplasmic and nuclear fractions as previously described [38]. About 20 μg of total extract, 10 μg of nuclear and 30 μg of cytoplasmic extracts (1:3 rate) were separated by SDS-PAGE and subjected to immunoblot.

Proteins were visualized by enhanced chemiluminescence (ECL, GE-Healthcare, Chicago, IL, USA) and ChemiDoc TM XRS system and analyzed by Quantity One W software (Bio-Rad, Milan, Italy).

### 2.10. Co-Immunoprecipitation

For co-immunoprecipitations (Co-IP) 2 × 10^6^ HEK293T cells were seeded in poly-D-lysine pre-treated 100-mm dishes; the day after cells were treated with sodium arsenite (Na Ars) (Sigma-Aldrich, Saint Louis, MO, USA) 250 μM for 30 min; cells extracts were incubated with an anti-PABP1 antibody (Sigma-Aldrich, Saint Louis, MO, USA), 3 μg for 1 mg of protein extract overnight at 4 °C. The day after, Dynabeads Protein G (Invitrogen, Carlsbad, CA, USA) were added to samples for 4 h, at 4 °C in rotation. Immunoglobulin G (IgG) 3 μg for 1 mg of protein extract was used as a negative control. Immunocomplexes were resolved with SDS-PAGE; immunoblot was performed with anti-YB-1 antibody (Abcam, Cambridge, UK).

### 2.11. Trichloroacetic Acid Precipitation

HEK293T cells were grown as previously described; Then, 24 h after seeding cells were gently rinsed twice with 1× PBS, and serum starved for 2 h. After treatments, cell culture medium was filtered through 0.22 μm filters to remove floating cells and debris. Scepter 2.0 (Millipore, Burlington, MA, USA) was used to determine if cells were still present in the collected medium. Trichloroacetic acid 25% was added to culture medium in 1:1 ratio, tubes were placed on ice for 30 min. Samples were then centrifuged at maximum speed at 4 °C for 30 min. Supernatant was discarded and cold acetone 100% was added to tubes (200 μL per sample), samples were then centrifuged at maximum speed, for 15 min, at 4 °C. This passage was done twice. Acetone was gentle removed and 200 μL of diethyl ether was added to tubes for 15 min on ice. After a centrifuge at maximum speed at 4 °C for 15 min, samples were placed on the block heater in order to evaporate diethyl ether. After addition of sample buffer. Samples were denatured in sample buffer (Sigma Chemical Co, St. Louis, MO, USA) and loaded on the gel.

### 2.12. Quantitative Real Time-PCR

A total of 3 × 10^5^ cells was seeded and let grow for 24 h; the day after, they were treated with recombinant YB-1 (rYB-1) or CCM-YB-1 for 24 h or left untreated.

For real time (RT)-PCR cells analysis, total RNA was isolated using the RNA Extraction Kit from Qiagen (Hilden, Germany) according to the manufacturer’s instructions. RNA (2–5 μg) was treated with DNAse I (Promega, Madison, WI, USA) and used to generate reverse transcribed cDNA using SuperScript III (Life Technologies, CA, USA), according to the manufacturer’s instructions. RT-PCR with SYBR green (Promega) detection was performed with a 7500 RT-PCR Thermo Cycler (Applied Biosystem, Foster City, Carlsbad, CA, USA). The relative quantification in gene expression was determined using the 2^−ΔΔCt^ method.

Expression levels were normalized using *g6pd* mRNA expression.

For each gene, primer sequences are presented in Supplementary Materials Table S3.

### 2.13. Statistical Analysis

Statistical analyses were performed using GraphPad Prism (version7.0, GraphPad Software Inc., San Diego, CA, USA).

Statistical significance of the difference in measured variables between control and treated groups was determined by *t*-test or analysis of variance (ANOVA) followed by Tukey’s or Dunnett’s multiple comparisons post-test. Difference were considered significant at *p* < 0.033 (*), *p* < 0.002 (**) and *p* < 0.001 (***). To report *p*-values, the New England Journal of Medicine (NEJM) decimal format was used; differences were considered statistically significant at * *p* < 0.033, ** *p* < 0.002 and *** *p* < 0.001. Detailed statistical information is shown in Table S2.

## 3. Results

### 3.1. YB-1 Is Recruited in Stress Granules under Diverse Stress Stimuli

**Figure 1:**
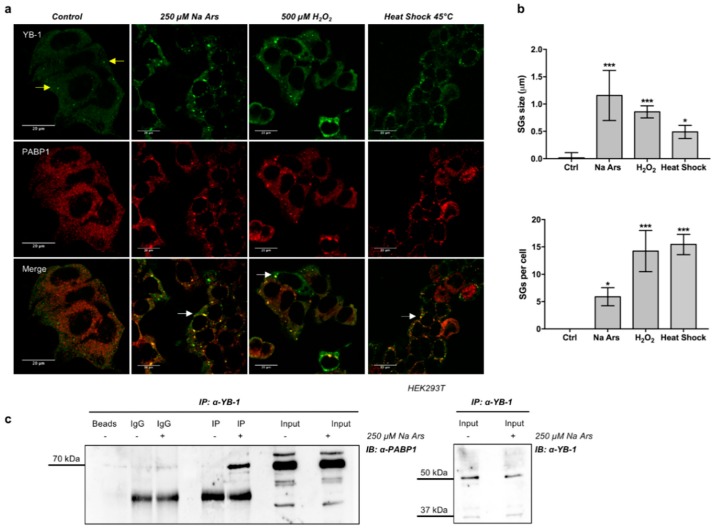
**Y-box-binding protein 1** (YB-1) and Poly(A)-binding protein 1 (PABP1) co-localize and interact in stress granules (SGs) under stress conditions. (**a**) Confocal immunofluorescence of un-treated (control) HEK293T, and treated with 250 µM sodium arsenite (Na Ars) for 30’, 500 µM H_2_O_2_ for 1 h or subjected to heat shock at 45 °C for 1 h, stained with α-YB-1 (green) and α-PABP1 (red); yellow and white arrows indicate P-bodies and stress granules respectively; (**b**) (Upper panel) size and number (lower panel) of SGs after treatments compared to control; statistical analysis was performed using 1-way ANOVA followed by Dunnett’s multiple comparisons test. Levels of significance are indicated (*** *p* < 0.001, * *p* = 0.001, see also Supplementary Materials Table S2); (**c**) Co-immunoprecipitation of HEK293T total protein extracts treated (+) or not (−) with 250 µM Na Ars for 30’; extracts were immunoprecipitated for YB-1 and immunorevealed for PABP1. Input samples immunorevealed with α-PABP1 and α-YB-1 are shown. Each panel is assembled from cropped Western blotting images (see original Western blot file for the original images).

It is documented that the functions played by YB-1 are strictly dependent on its subcellular localization [16,39,40]. Thus, we examined YB-1 subcellular localization in human HEK293T cells in resting conditions and following treatment with Na Ars, a well-known inducer of oxidative stress and translational arrest [41,42]. By double immunofluorescence labelling and confocal microscopy using antibodies against YB-1 and PABP1, another specific SGs marker [43], we found that YB-1 and PABP1 were evenly distributed in the cytoplasm in resting conditions (Figure 1a, control). However, following heat shock or treatment with Na Ars or hydrogen peroxide, YB-1 was found to co-localize with PABP1 in cytoplasmic stress granules (Figure 1a). Interestingly, the size and overall number of SGs per cell were different depending on the type of stimulus applied (Figure 1b, upper and lower panels), thus confirming previous findings indicating stress-specific differences in composition and assembly of stress granules [44].

Next, we immunoprecipitated YB-1 protein from extracts of HEK293T cells left untreated (−) or treated with 250 μM Na Ars (+). As shown in Figure 1c, PABP1 was detectable in YB-1 immunocomplexes exclusively from Na Ars treated cells, indicating that YB-1 and PABP1 association occurs predominantly in SGs.

**Figure 2:**
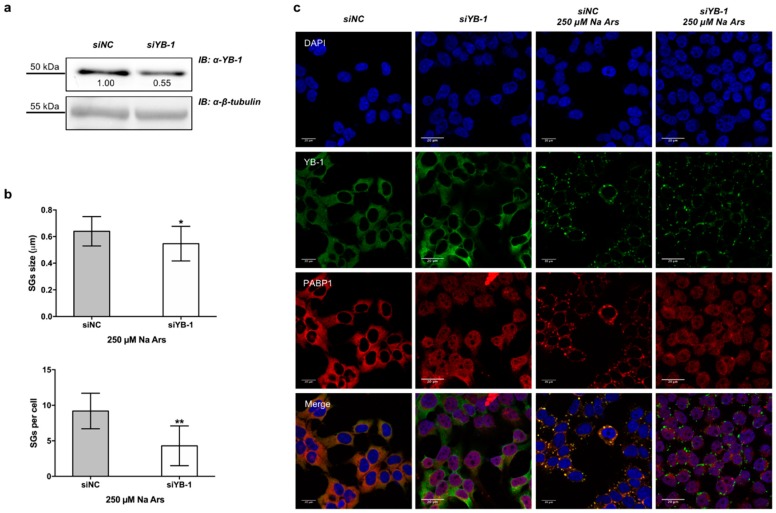
Silencing of YB-1 affects PABP1 positive SGs formation. (**a**) Western blot of total extract from control (siNC) or 100 nM YB-1 silenced (siYB-1) HEK293T; the degree of reduction of YB-1 protein levels in YB-1 siRNA-treated cells compared with control is indicated beneath each band in the Western blot (where the relative unit 1.0 represents YB-1 levels in cells transfected with the control siRNA). β-tubulin was used as loading control. Each panel is assembled from cropped Western blotting images (see original Western blot file for the original images); (**b**) (Upper panel) size and number (lower panel) of stress granules (SGs) in YB-1 silenced cells treated with Na Ars compared to siNC (control) cells; statistical analysis was performed using unpaired *t*-test with Welch’s correction (** *p* = 0.010 and * *p* = 0.02 see Supplementary Materials Table S2); (**c**) Confocal immunofluorescence of control (siNC) and silenced (siYB-1) HEK293T cells, treated or not with 250 µM Na Ars for 30’, stained with α-YB-1 (green) and α-PABP1 (red), nuclei are stained with DAPI (blue).

To determine the relevance of YB-1 in SGs assembly, we depleted HEK293T cells of YB-1 using a specific siRNA pool against endogenous YB-1 mRNA (siYB1). By immunoblot and densitometric analysis we found that the expression level of YB-1 protein was reduced to 55% of control (Figure 2a). However, YB-1 knock-down consistently impaired the assembly of arsenite-induced PABP1-positive stress granules by reducing their size and number (Figure 2b, upper and lower panels and Figure 2c). Interestingly, in YB-1 depleted cells, PABP1 was located into the nucleus both in resting and under stress condition (Figure 2c), thus suggesting that YB-1 may act as a cytoplasmic anchor for PABP1.

### 3.2. Arsenite-Induced Oxidative Stress Promotes YB-1 Secretion

**Figure 3:**
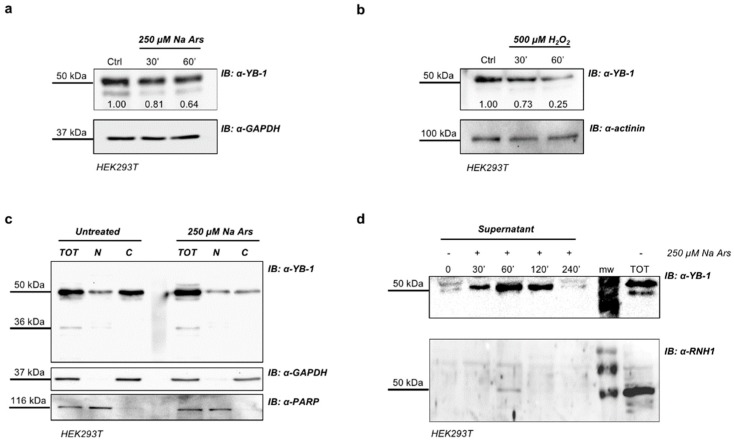
Na Ars induced oxidative stress promotes YB-1 secretion. Western blot of total protein extracts from HEK293T treated with 250 µM Na Ars (**a**) or 500 µM H_2_O_2_ (**b**) for 30’ and 60’; glyceraldehyde 3-phosphate dehydrogenase (GAPDH) and actinin were used as loading control. The degree of reduction of YB-1 protein levels in treated cells compared with controls is indicated beneath each band in the Western blot (where the relative unit 1.0 represents YB-1 levels in control cells); (**c**) Western blot of total extract and nuclear/cytoplasmic fractionation of Na Ars treated and untreated HEK293T. GAPDH and PARP were used as loading control for cytoplasm and nucleus, respectively; (**d**) Western blot of TCA precipitated supernatants of HEK293T cells. Ribonuclease/Angiogenin Inhibitor 1 (RNH1) was used as negative control of cell lysis. Each panel is assembled from cropped Western blotting images (see original Western blot file for the original images).

We performed Western blotting analysis to reveal YB-1 protein in total extracts of Na Ars and H_2_O_2_-treated HEK293T cells. Despite the apparent enrichment of YB-1 in SGs, we found a significant reduction of the level of intracellular YB-1 protein (Figure 3a,b). By immunoblot analysis of HEK293T nuclear and cytoplasmic fractions we found that the reduction of YB-1 protein level induced by Na Ars was exclusively at the expense of the cytoplasmic pool, while nuclear YB-1 was totally unaffected (Figure 3c). YB-1 was previously found to be secreted [45,46] therefore we hypothesize that the decrease of cytoplasmic YB-1 was due to oxidative stress-enhanced YB-1 secretion. To prove this hypothesis, we treated HEK293T cells with Na Ars and precipitated the supernatant by TCA at different time points (from 30’ to 240’). Trichloroacetic acid-precipitated fractions were then subjected to immunoblot and revealed with antibodies against YB-1 and Ribonuclaese/Angiogenin Inhibitor 1 (RNH1) a not secreted cytoplasmic ribonuclease inhibitor abundantly expressed in HEK293T [47]. As shown in Figure 3d, in the 240’ time course the amount of secreted YB-1 protein upon Na Ars treatment significantly increased and peaked after 60’. RNH1 signal was barely detectable ensuring that the increase of exYB-1 was not due to cell death and passive release (Figure 3d, lane 3, lower panel). Moreover, after 30’ and 60’ treatment with 250 μM Na Ars or 500 μM hydrogen peroxide, cell viability was only slightly reduced (90% and 94%, respectively) compared to control cells and the increase of trypan blue positive cells was negligible (Figure S1a,b, respectively). These data further indicate that extracellular accumulation of YB-1 was not due to cell injury.

Similar results were obtained from Western blot analysis of supernatants from HEK293T cells transfected with Myc-YB-1 expression plasmid and treated with Na Ars 250 μM for 30’, 60’ and 120’. Immunoblotting of TCA-precipitated CCM media showed the presence of Myc-tagged YB-1 only in arsenite-stimulated cells thus indicating that stressed cells can also secrete transfected YB-1 (Figure S2).

### 3.3. Extracellular YB-1 Exerts Anti-Proliferative Effects on Receiving Cells

To analyze the effects of exYB-1on receiving cells we prepared a YB-1 enriched fraction from HEK293T conditioned cell culture medium (CCM-YB-1) by ammonium sulphate precipitation followed by high performance liquid chromatography (HPLC) purification. Furthermore, we produced a recombinant YB-1 from *Escherichia coli*, that was accumulated mainly in bacterial cytosolic fraction. CCM-YB-1 and rYB-1 were analyzed by SDS-PAGE and YB-1 immunopositive bands were manually excised as reported in Figure S3. Each band was subjected to in situ tryptic digestion and peptides eluted from the gel were analyzed by LTQ-Orbitrap mass spectrometer (Thermo-Scientific, Waltham, MA, USA) (see Appendix A). By matching MS and MSMS data obtained from the mass spectrometer and peptide sequence databases by Mascot search engine, a high number of identified peptides was obtained with an error lower than 10 ppm. We obtained a high sequence coverage of 79% for R1 band (rYB-1 band 1 in Figure S3), 78% for R2 (rYB-1 band 2 in Figure S3), 60% for S1 and 29% for S2 (CCM-YB-1 band 1 and 2 respectively, in Figure S3). A list of peptides with relative score, sequence and modification allowed to unambiguously identify the YB-1 protein with a high sequence coverage and protein score (Table S1).

We have previously demonstrated that intracellular YB-1 is implicated in keratinocytes proliferation and survival to oxidative stress [40,48]. To get an insight into the function of secreted YB-1 we evaluated the proliferative response of HaCaT cells to the addition of rYB-1 or HPLC-purified YB-1 from HEK293T cell culture medium (CCM-YB-1).

**Figure 4:**
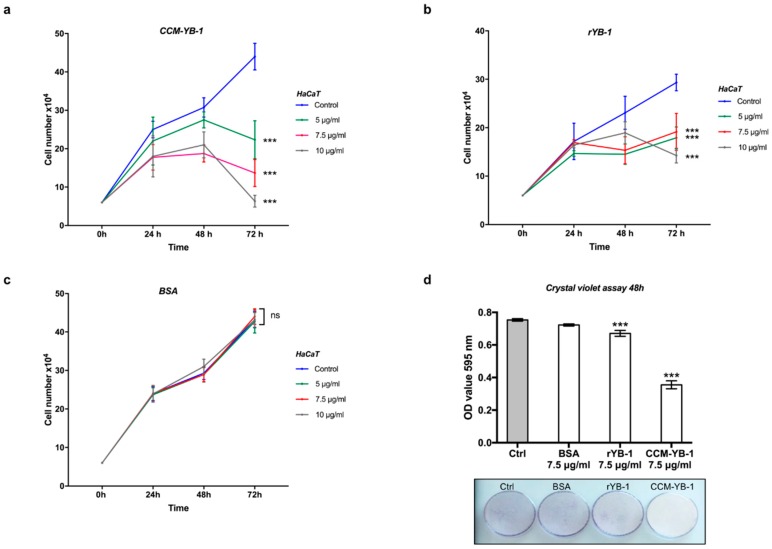
Extracellular YB-1 (exYB-1) affects HaCaT cell proliferation. Cell proliferation profile of HaCaT cells incubated with indicated concentrations of CCM-YB-1 (**a**), rYB-1 (**b**) or Bovine Serum Albumin (BSA) (**c**); statistical analysis was performed using 2-way ANOVA followed by Dunnett’s multiple comparisons test. Levels of significance are indicated (*** *p* < 0.001, see also Supplementary Materials Table S2); (**d**) Crystal violet assay of HaCaT cells treated with 7.5 μg/mL of CCM-YB-1, rYB-1, BSA or left untreated (Control; (bars) optical absorbance at 595 nm is reported on the y-axis; (image) representative colorimetric evaluation. Statistical analysis was performed using 1-way ANOVA followed by Dunnett’s multiple comparisons test. Levels of significance are indicated (*** *p* < 0.001, see also Supplementary Materials Table S2).

HaCaT cells were then incubated for the indicated time with increasing amounts (5.0, 7.5 and 10 μg/mL) of rYB-1 protein, CCM-YB-1 or Bovine Serum Albumin (BSA) as control in serum supplemented culture medium. As shown in Figure 4a,b, treatment with CCM-YB-1 or rYB-1 reduced the rate of proliferation of HaCaT cells while equivalent amounts of BSA were ineffective (Figure 4c). This effect was not cell type specific since it was also observed using HEK293T and CaCo2 as receiving cells (Figure S4). Remarkably, compared to rYB-1, CCM-YB-1 exerted a stronger inhibitory effect on all cell lines tested (Figure 4a,b; Figure S4). This was confirmed by Crystal Violet Assay (CVA) where HaCaT cells treated with 7.5 μg/mL of CCM-YB-1 for 48 h showed a 47% reduction in viable cells compared to a 11% reduction obtained with the same amount of rYB-1 (Figure 4d).

**Figure 5:**
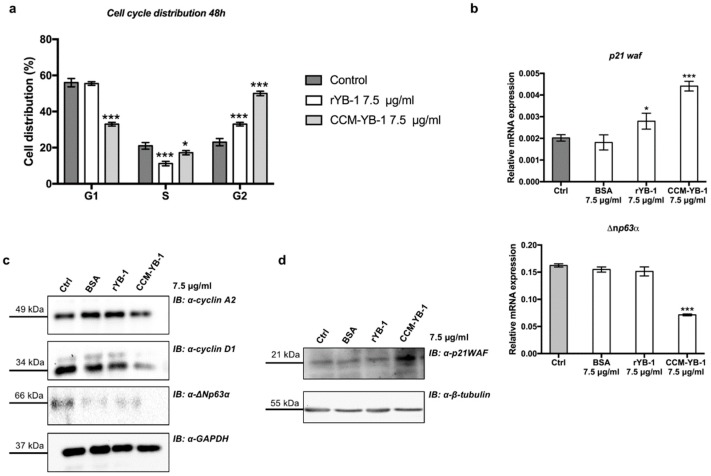
Extracellular YB-1 induced G2/M phase arrest. (**a**) Cell cycle profile of HaCaT cells treated with 7.5 μg/mL of CCM-YB-1, rYB-1, BSA or left untreated for 48 h. Statistical analysis was performed using 2-way ANOVA followed by Dunnett’s multiple comparisons test. Levels of significance are indicated (*** *p* < 0.001, * *p* = 0.01, see also Supplementary Materials Table S2); (**b**) RT-qPCR analysis of *p21waf* and *∆np63α* in HaCaT cells treated with 7.5 μg/mL of CCM-YB-1, rYB-1, BSA or left untreated (Control for 48 h. Statistical analysis was performed using 1-way ANOVA followed by Dunnett’s multiple comparisons test. Levels of significance are indicated (*** *p* < 0.001, * *p* = 0.01, see also Supplementary Materials Table S2); (**c**,**d**) Western blot analysis of total extracts of HaCaT cell treated with rYB-1 and CCM-YB1 and revealed with the indicated antibodies. GAPDH and β-tubulin were used as loading control. Each panel is assembled from cropped Western blotting images (see original Western blot file for the original images).

The effect on the rate of cell proliferation caused by CCM-YB-1 and rYB-1 was associated to a G2/M phase cell cycle arrest, as indicated by flow cytometry analysis of HaCaT cells treated with exYB-1 (Figure 5a). Finally, to explore the molecular mechanism underlying inhibition of cell proliferation by exYB-1we analyzed by quantitative PCR the mRNA level of *p21waf* and *∆np63α* in rYB-1 or CCM-YB-1 treated HaCaT cells. p21WAF is a relevant cell cycle marker that induces G1 and G2/M cell cycle arrest by inhibiting the kinase activity of Cyclin-dependent kinase (CDK)-cyclin complexes [49] while ∆Np63α maintains the proliferative capacity of keratinocytes [50]. *p21waf* was strongly induced by both rYB-1 and CCM-YB-1 while *∆np63α* was significantly downregulated only by treatment with CCM-YB-1 (Figure 5a,b). By Western blot analysis we also confirmed reduction of ∆Np63α and induction of p21WAF at protein level (Figure 5c,d). Moreover, according to the observed cell cycle arrest the level of cyclin A2 and cyclin D1 were also reduced (Figure 5c).

## 4. Discussion

Although YB-1 was originally identified as a transcription factor belonging to the Y-box binding family, a number of works provide evidence that YB-1 possesses additional functions, independent of its DNA binding activity [7,9,39]. Based on more recent literature, a possible regulatory role of YB-1 in inflammation and stress response has started to emerge [4].

The goal of our study was to investigate the function of YB-1 protein under stress conditions. In unstressed cells, YB-1 was diffusely distributed throughout the cytoplasm. Upon addition Na Ars, H_2_O_2_ and heat shock at 45 °C, we observed YB-1 protein association to SGs, which constitute a canonical cellular response to stress. In HEK293T cells, YB-1 almost complete co-localizes with the SGs marker PABP1; the co-localization was accompanied by the physical interaction between the two proteins, only in stress conditions.

Moreover, we show that YB-1 silencing impairs the assembly of PABP-1 positive SGs under stress conditions, thus confirming that YB-1 plays a pivotal role in the aggregation of SG components. Interestingly, we also found that PABP1 was mainly nuclear in YB-1 depleted cells, under normal as well as stress conditions, indicating that YB-1 may retain PABP1 protein in the cytoplasmic compartment. This observation raises the question of whether YB-1 silencing, by altering PABP1 localization, also influences the metabolism of PABP1-associated transcripts.

Involvement of YB-1 in SGs formation has potential pathological relevance in vivo, given that SGs formation in cancer cells might protect them against stress-induced cell damage and death [2]. Indeed, because of metabolic and signaling aberrations, cancer cells exhibit elevated ROS levels that can promote tumor formation and progression by inducing DNA mutations and pro-oncogenic signaling pathways [51]. By impairing SGs assembly, YB-1 depletion may weaken the antioxidant capacity of tumor cells, thus sensitizing them to necrosis/apoptosis and may have a therapeutic impact in cancer chemotherapy.

In the process of characterization of YB-1 response to cellular stresses, we observed a decrease of total amount of the protein after Na Ars or hydrogen peroxide treatments. This result sounded unexpected, considering the relevance of YB-1 in SGs.

Remarkably, the decrease of YB-1 protein level was at the expenses of cytoplasmic YB-1, while nuclear YB-1 was totally unaffected.

However, considering that YB-1 is normally released in the culture medium by HEK293T cells [28], we speculated that stress stimuli may function as enhancers of YB-1 protein secretion.

Interestingly, we found a significant increase of exYB-1 level during Na Ars treatment; such increase was clearly detectable after 30 min and peaked at 60 min of treatment. After 4 h of treatment the level of exYB-1 was reduced probably because of protein degradation. It should be noticed that non-specific YB-1 release due to cell death was excluded by cell viability assays (viability of treated cells >95%) and the lack of comparable RNH1 release, a highly abundant cytoplasmic protein.

YB-1 secretion after cell stimulation is not unprecedented. Recently, YB-1 was found to be secreted by Lipopolysaccharide (LPS)-stimulated human monocytes and mesangial cells and acts as an extracellular mitogen by interacting with Notch-3 receptor [45]. YB-1 has been isolated in extracellular vesicles and postulated to play a role in sorting small noncoding RNAs into a subpopulation of exosomes [52]. However, it has to be noted, that in previous manuscripts neither YB-1 positive SGs formation nor reduction of intracellular YB-1 was observed in stimulated cells [45].

Extracellular occurrence of YB-1 raises the question of its functional relevance. So far, the functional role of exYB-1and the pathological implications of secreted YB-1 are largely unknown. Data from other laboratories indicated that exYB-1may be involved in tumor progression since addition of recombinant YB-1 to cancer cell lines revealed pro-mitogenic effects [45].

Intriguingly, we found that exYB-1exerts an anti-proliferative effect on receiving HaCaT, HEK293T, and CaCo-2 cells. Remarkably, YB-1 is normally released in the culture medium by HEK293T cells but not by HaCaT or CaCo2 cells even though all these cell lines express high levels of endogenous YB-1. This may depend on a particular pathway constitutively activated in HEK293T cells or rely on specific posttranslational modifications occurring in HEK293T and possibly other cell contexts facilitating YB-1 extracellular release.

Treatment of HaCaT cells with exYB-1induced a G2/M cell cycle arrest associated with p21WAF induction and ∆Np63α reduction. The effect of CCM-YB-1 was more pronounced compared to that of the rYB-1. We can speculate that specific posttranslational modifications, such as ubiquitination and/or sumoylation, occurring in mammalian cells can explain the different effects showed by CCM-YB1 and rYB-1 proteins on receiving cells. Alternatively, bacterially synthesized rYB-1 may have a different protein folding that partially affect its functions.

It has been reported that intracellular YB-1 activates Notch signalling [53] and acts as a mediator of signals transmitted via the EGFR-RAS-MAPK [54] enhancing ∆Np63α level in keratinocytes. Therefore, we postulate that exYB-1may either activate Notch or repress EGFR pathways, thus reducing ∆Np63α level which is known to support the proliferative potential of normal and transformed keratinocytes [55].

We can presume that upon severe tissue-injury, exYB-1can signal to neighboring cells that a cell cycle arrest is required to allow cells to recover from stress and/or avoid the propagation of genetic lesions and repair them. However, further studies are needed to define the molecular mechanisms by which YB-1 can signal and regulate this response in receiving cells and the exact cellular source and targets in patients with malignant disorders.

## Supplementary Materials

The following are available online at http://www.mdpi.com/2073-4425/9/10/513/s1, Figure S1: YB-1 presence in the cell medium is not due to cell death and lysis, Figure S2: Transfected myc-YB-1 can be secreted by cells, Figure S3: Band excision, Figure S4: Extracellular YB-1 affects HEK293T and CaCo2 cell proliferation. Table S1: MSMs analysis or rYB-1 and CCM-YB-1, Table S2: Statistical analysis, Table S3: RT-qPCR assay PCR primers.

## Appendix A

### Appendix A.1. Preparation of Bacterial Expression Vector pET22b^(+)^/YB-1

Recombinant vector pEX-A2 containing human YB-1 cDNA sequence (purchased from Eurofins Genomics, Edersberg, Germany), has been used as template for a PCR reaction.

PCR has been carried out using following primers: forward 5′-CC**CATATG**AGCAGCGAGGCCGAGACCC-3′; reverse 5′-GG**AAGCTT**GCCTCGGGAGCGGGAA-3′. Bold and underlined sequences refer to restriction sites (NdeI and HindIII), designed to permit the insertion of YB-1 coding sequence upstream to a His-Tag sequence to facilitate the purification of the recombinant protein. The PCR reaction was carried out in a total volume of 50 μL containing 60 ng of each primer, 20 ng of template, deoxyribonucleotides (dNTPs) containing 0.2 mM of each dNTP, 5 μL 10× Taq buffer and 1.5 units of Taq DNA polymerase (EuroClone, Milan, Italy). PCR was performed under the following conditions: An initial denaturation step at 98 °C for 5 min, followed by 38 cycles of 30 s at 98 °C, 1 min at 65 °C, and 2 min at 72 °C. PCR products were then isolated by electrophoresis on 1% agarose gel, and purified using the Product Purification Kit (Roche Applied Science, Monza, Italy). Purified DNA, treated with NdeI and HindIII restriction enzymes, was inserted into the pET22b (+) expression vector by ligation reaction catalyzed by T4 DNA ligase (Thermo Fisher Scientific, Waltham, MA, USA). All cloned, purified DNAs were certified through sequencing (Eurofins Genomics, Edersberg, Germany) before processing. The recombinant expression plasmid was used then to transform competent *E. coli* strain BL21(DE3) (New England BioLabs, Ipswich, MA, USA).

### Appendix A.2. Recombinant YB-1 Protein Preparation

The expression plasmid pET22b(+)/YB-1 was used to transform competent *E. coli* strain BL21(DE3) cells. Cells were grown at 37 °C to an *A*_600 nm_ = 0.7, and then induced with 0.6 mM isopropyl-1-thio-d-galactopyranoside (IPTG) and grown overnight. The expression level of rYB-1 was estimated performing a Western blot on two bacterial aliquots collected before the addiction of IPTG and after 16 h respectively. Bacterial cells were then harvested by centrifugation (7000 rpm, 4 °C, 15 min), re-suspended in 100 mM Tris acetate buffer (pH 8.0) and subjected to sonication in presence of protease inhibitors. Cell debris and precipitated proteins were removed by centrifugation (17,500× *g*, 1 h) whereas the supernatant was subjected to at least three washes in 100 mM Tris acetate pH 8.0 and finally centrifuged to separate inclusion bodies and cytosolic soluble fraction, the latter containing rYB-1. Recombinant YB1 was purified performing a two-step procedure composed by an affinity chromatography followed by a reverse phase chromatography.

### Appendix A.3. Chromatographic Procedures

Affinity chromatography was carried out on HisTrap resin (GE Healthcare, Chicago, IL, USA) equilibrated with 100 mM Tris acetate pH 8.0 containing 20 mM imidazole. Elution was carried out by using 100 mM Tris acetate pH 8.0 containing 500 mM imidazole and spectrophotometrically monitored at 280 nm. Reverse phase chromatography was performed on a HPLC device Series 200 (Perkin Elmer, Waltham, MA, USA) by using a C4 column (Dr. Maisch GmbH, Ammerbuch-Entringen, Germany), 0.1% TFA in 5% acetonitrile as buffer A and 0.1% TFA in 95% acetonitrile as buffer B. Elution was performed by setting a linear gradient 5–95% buffer B in 60 min and spectrophotometrically monitored at 278 nm.

### Appendix A.4. Extraction of Human Secreted YB-1 from HEK293T Cell Culture Medium

All culture media, enriched with secreted YB-1, have been processed with three subsequent steps of protein precipitations by using ammonium sulfate (Sigma Aldrich, Milan, Italy). The concentration of ammonium sulfate was increased from 0 to 30%, 30% to 70% and, finally until 100%, determining proteins salting out. Between each step, media were centrifuged 1 h at 14,000 rpm and 4 °C to sediment protein aggregates. All sediments were finally re-suspended in PBS, quantified and then analyzed by SDS-page and Western blotting.

### Appendix A.5. Protein Separation by SDS-PAGE and In Situ Digestion

The proteins precipitated from culture medium were separated by 12% SDS-PAGE, under reducing conditions. Proteins were visualized by colloidal Coomassie brilliant blue and the immunopositive bands were excised, also depending on the visualization of bands at expected molecular weight. Each band was subjected to in situ digestion protocol by reducing proteins by 10 mM DTT and alkylation with 55 mM iodoacetamide, according to the established protocol. Finally, each dry gel piece was rehydrated in 40 μL of 10 mM ammonium bicarbonate solution containing 200 ng of Trypsin Gold, Mass Spectrometry grade (Promega, Madison, WI, USA), and incubated at 37 °C overnight. The trypsinolysis was stopped with 0.1% formic acid (FA), and tryptic peptides were eluted, vacuum dried, and dissolved in 0.1% formic acid for LC-MS/MS analysis.

### Appendix A.6. LTQ-Orbitrap Analysis

Peptides were separated on a 1.7-μm BEH C18 column (Waters Corporation, Milford, MA, USA) at a flow rate of 280 nL/min by using as mobile phase solution A: 95% water, 5% acetonitrile, 0.1% acetic acid; solution B: 95% acetonitrile, 5% water, 0.1% acetic acid. Peptide elution was achieved along a linear gradient of B from 20% to 90% over 53 min. MS and MS/MS data were acquired on an Orbitrap LTQ XL high-performance liquid chromatography MS system (Thermo-Scientific, Waltham, MA, USA) equipped with an electrospray source (ESI). The full scan precursor MS spectra (400–1600 *m*/*z*) were acquired in the Velos-Orbitrap analyzer with a resolution of *r* = 60,000. This was followed by data dependent MS/MS fragmentation in centroid mode of the most intense ion from the survey scan using collision-induced dissociation (CID) in the linear ion trap: Normalized collision energy 35%; activation Q 0.25; electrospray voltage 1.4 kV; capillary temperature 200 °C; and isolation width 2.00. The targeted ions were dynamically excluded for 30 s, and this MS/MS scan event was repeated for the top 20 peaks in the MS survey scan. The five most intense double and triple charged peptide ions were selected and fragmented. The resulting MS data were processed by MS Converter General User Interface software 3.0 (ProteoWizard) to generate peak lists for protein identifications. Database searches were carried out on the Mascot Deamon version 4.1 by Matrix Science (London, UK). The SwissProt database (release January 2017, 547,599 sequences; 195,014,757 residues) was employed (settings: two missed cleavages; carbamidomethyl (C) as fixed modification and Oxidation (M), Phospho (ST), Phospho (Y), Acetyl (K), Gln->pyro-Glu (N-term Q), Glu->pyro-Glu (N-term E) as variable modifications; peptide tolerance of 10 ppm and fragment mass tolerance of ±0.6 Da).
